# Sliding Interaction for Coated Asperity with Power-Law Hardening Elastic-Plastic Coatings

**DOI:** 10.3390/ma12152388

**Published:** 2019-07-26

**Authors:** Bin Zhao, Hanzhang Xu, Xiqun Lu

**Affiliations:** College of Power and Energy Engineering, Harbin Engineering University, Harbin 150001, China

**Keywords:** sliding, coated asperity contact, contact force, friction coefficient, power-law hardening materials

## Abstract

Sliding between asperities occurs inevitably in the friction pair, which affects the efficiency and reliability in both lubricated and non-lubricated conditions. In this work, the contact parameters in the coated asperity sliding process are studied, and the universal expressions of the average contact force and the friction coefficient are obtained. The effect of the interference between asperities, the material and geometrical parameters including the Young’s modulus ratio and yield strength ratio of the coating and substrate, and the hardening exponent and thickness of the coating on the average contact forces and friction coefficient is considered. It shows both normal and tangential contact forces increase with the increasing interference, increasing Young’s modulus ratio, decreasing yield strength ratio, and decreasing coating thickness; while the trend is different for the effect of the hardening exponent of the coating. The normal force increases and the tangential force decreases as the hardening exponent increases. Based on this, the influence of these parameters on the effective friction coefficient is obtained further. It reveals that the friction coefficient increases as the interference and Young’s modulus ratio enlarge and decreases as the yield strength ratio, the coating’s hardening exponent, and thickness increase. The universal expressions for the contact force and friction coefficient in the sliding process are obtained. This work might give some useful results to help choose the optimum coatings for specific substrates to reduce friction in cases where the asperity contact exists, especially in the focused field of the journal bearing in the marine engine under poor lubrication conditions.

## 1. Introduction

In applications where relative movement exists, sliding between surfaces affects mechanical performance [1] or might cause fault in components and parts of different machines [2,3]. From a micro perspective, engineering surfaces are not absolutely smooth but usually contain many asperities. Actually, the sliding between surfaces could be regarded as the relative movement between the asperities on different surfaces. That is to say, sliding between asperities is a foundational topic.

Coatings are widely used in industry applications to improve the tribological performance of the surfaces, e.g., to reduce friction and enhance wear resistance [4,5]. For example, in the field of the main bearing bush in marine engines, where the authors focused, the babbitt metal is widely used on the steel back, which could be seen as a kind of coating in some sense. Finding out an effective method to choose the optimal coating under specific conditions is relevant but also challenging. For now, the most common approach is still the trial and error method as far as the authors know. It would be helpful to develop more effective coating selection methods by enriching the relevant fundamental theory about the frictional behavior of the coated surfaces, which is the direction of the authors’ efforts. In this work, the coated asperity sliding interaction is considered as a foundational frictional problem between the coated surfaces, and the effect of the coating parameters including the geometrical and material parameters on the contact parameters is explored.

The sliding process between asperities has been considered by some researchers so far. Several methods were generally used to study this process, including the experimental approach and the theoretical approach. Some experimental works, which studied the sliding of the single asperity in the micro scale, were accomplished with apparatuses such as the atomic force microscope [6,7] or the scratch tester in micro or nano scale [8,9]. These studies have high requirements for experimental equipment, thus, many other researchers selected the theoretical way, such as the analytical method and the numerical method. Initially, the analytical method was adopted to study the elastic contact of the asperity suffering the normal and tangential loads as given by Cattaneo [10] and Mindlin [11]. Keer et al. [12] studied the tangential contact between two objects with different material and geometrical parameters. Hamilton [13] considered the sliding process and obtained the analytical equations of tensile stresses and yield parameters. However, the analytical method showed some limitations when some complex contact cases were studied with complex boundary conditions and material attributes. As computation power developed, the numerical method was more and more widely used by the researchers. The molecular dynamics method is an atomistic simulation to explain some phenomena in nature. Yang and Shi [14] explored the wear phenomenon in the frictional sliding process between an asperity and a rigid flat with the molecular dynamic simulation considering the generation of debris. Vadgama et al. [15] investigated the dry sliding between copper asperities and considered the effect of the interference, the sliding velocity, radius of the asperity, and the lattice orientation on the contact parameters with the molecular dynamic method. However, the foundation of the molecular model is still difficult, especially for complex physical materials and the computational process is very time consuming. By comparison, the finite element method was another widely used numerical approach. Faulkner and Arnell [16] gave a finite element model to study the sliding process between two homogeneous elastic-plastic asperities, and to explore the evolution rule of the contact force during the sliding process. A similar but more detailed model was developed by Vijaywargiya and Green [17] to consider the change of the contact stresses and the energy loss besides the contact forces in the sliding interaction. Jackson et al. [18], and later Zhao et al. [19], developed similar semi-analytical models considering different homogeneous asperity materials. In their works, sliding was regarded as lots of tangential loading–unloading processes between two asperities, and the universal equations of the contact parameters about the tangential loading and unloading obtained previously could be adopted. The semi-analytical results were compared and validated with the finite element results. The empirical equations of the contact force and the friction coefficient caused by the plastic deformation in the sliding process were obtained then. Mulvihill et al. [20] also studied the homogeneous asperity sliding process by considering more interference with adhesion and material failure, and the change of the friction coefficient was explored under different conditions. 

Most of the mentioned works about sliding interaction considered homogeneous asperities. For coated asperity contact, researchers also made some efforts. Dong et al. [21] and Chen et al. [22] developed a layered contact algorithm to study the contact between a rigid asperity and a coated elastic or elastic-plastic half space, which was solved by means of the Fast Fourier Transformation (FFT) technology. Recently, Wang and Schipper [23] used a similar method to study the sliding process of two elastic-plastic asperities with a nanocrystalline layer and tribofilms and to explore the effect of the mechanical properties of the tribofilm and the interference depth on the contact parameters. Most of these works considered the contact between a rigid asperity and an elastic or elastic–perfectly plastic half space. When the materials were expanded to include more actual elastic-plastic cases but not the ideal materials, this contact algorithm showed some limits. In the last two years, Chen et al. [24] studied the normal contact between a linear-hardening elastic-plastic coated asperity and a rigid flat in detail, to investigate the contact parameters including the stress and contact force in the coating and the substrate. In their works, the universal relation of the contact loads and the parameters of the asperity including the coating thickness, the Young’s modulus, yield strength, and Poisson’s ratio of the coating and substrate was given. In addition, they studied the static friction coefficient in full stick condition considering the contact between a rigid flat and an elastic-plastic coated asperity under combined normal and tangential loading, and the universal expressions of the static friction coefficient relating to the coating thickness, the normal loads, and the material properties of the coatings were given. 

However, for now, only a few works have considered the sliding process between coated asperities and given the general expression of the contact force and effective dynamic friction coefficient related to the geometrical and material parameters (especially for coated asperities with power-law hardening elastic-plastic coatings). Thus, it might be essential to explore the effect of the geometrical and material parameters of the coating on the contact properties and then to get the general expression of the contact parameters in the coated asperity sliding process. Compared with the trial and error way, that would help choose the optimum coatings for the specific substrate materials for the sake of the low-friction design. Moreover, as the previous studies indicated, the hardening behavior of the elastic-plastic asperity materials also showed some influence on the contact parameters in the sliding process between homogeneous asperities [19]. For coated asperity sliding interactions, the effect of the hardening behavior has not been investigated yet. In this work, the sliding between a coated asperity and a homogenous asperity is studied, which exists in some applications (e.g., the shaft and the bearing bush in the marine engine under very bad lubrication conditions, as mentioned before), and the effect of the material and geometrical parameters of the coated asperity on the contact force and friction coefficient is explored. Based on this, the universal expressions of the normal and tangential contact forces and the dynamic friction coefficients is derived. It should be noted that only the friction behavior (e.g., friction coefficient) due to the plastic deformation of the coated asperity in the sliding process is focused on. As indicated by Bosman and Schipper [25], the frictional behavior (e.g., mild wear) is related closely to the plastic deformation of the uppermost material in the poor lubrication condition, which means the relation between the frictional behavior and the plastic deformation of the coated asperity is essential to be studied. Thus, in this work, the frictionless contact condition is adopted, and adhesion and failure are ignored to focus on this relation between the frictional behavior (e.g., contact force and friction coefficient) and the plastic deformation.

## 2. Finite Element Model

**Figure 1:**
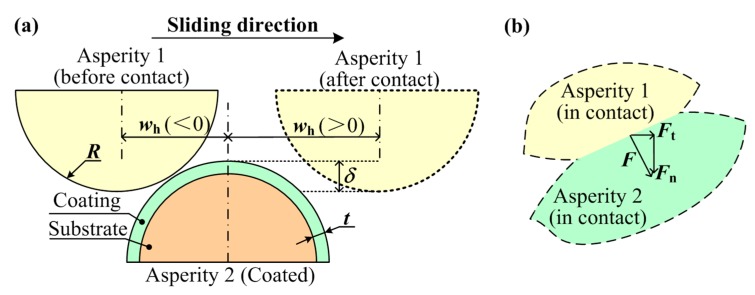
Schematic of the sliding process between two asperities. (**a**) Two asperities in sliding process, (**b**) the forces showing in different directions (including normal and tangential directions).

The sliding process between two asperities was considered in the micro scale, as shown in Figure 1. One asperity (Asperity 1) is assumed as homogeneous, and the other one (Asperity 2) is a coated asperity. The contact force, *F*, of the asperity in the sliding process could be decomposed as the normal and tangential components (*F*_n_ and *F*_t_). As in the field of the main bearing in the marine engine studied by the authors, the sliding process would happen between the bearing bush and the crankshaft especially in the poor lubrication or the solid-solid contact status such as the start-stop process, where about 40% of bearing failures occur. Thus, the frictional behavior should be focused on this process, and in this work, the research scope is limited to the solid-solid contact case. In the micro scale, the bearing bush including the steel back (the substrate) and the alloy layer (the coating) could be treated as the coated asperity, while the crankshaft could be seen as the homogeneous asperity. The material properties would be adopted as the crankshaft-bearing system in the marine engine. The crankshaft and steel back materials of the bearing are both steel materials, which could be set as the 2% linear hardening elastic-plastic material [26], while the coating (the commonly used babbitt metal SnSb11Cu6) could be set as the power-law hardening elastic-plastic materials [27]. The geometrical and material parameters are set as follows: The radius of the two asperities, *R*; the coating thickness, *t*, of Asperity 2; the interaction, *δ*, between the two sliding asperities; the horizontal coordinate, *w*_h_, of Asperity 1; the Young’s modulus, *E*_1_, the yield strength, *Y*_1_, and the Poisson’s ratios, *ν*_1_, of Asperity 1; the Young’s modulus, *E*_co_, the yield strength, *Y*_co_, the hardening exponent, *n*, and the Poisson’s ratios, *ν*_co_, of the coating of Asperity 2; and the Young’s modulus, *E*_su_, the yield strength, *Y*_su_, and the Poisson’s ratios, *ν*_su_, of the substrate of Asperity 2. The typical geometrical and material parameters were chosen as the values given by the marine engine manufacturer (listed in Table 1), including the dimensionless coating thickness, *t/R*, the dimensionless asperity interaction, *δ/R*, the Young’s modulus, the yield strength, the Poisson’s ratio, and the hardening type of the two asperities.

**Figure 2:**
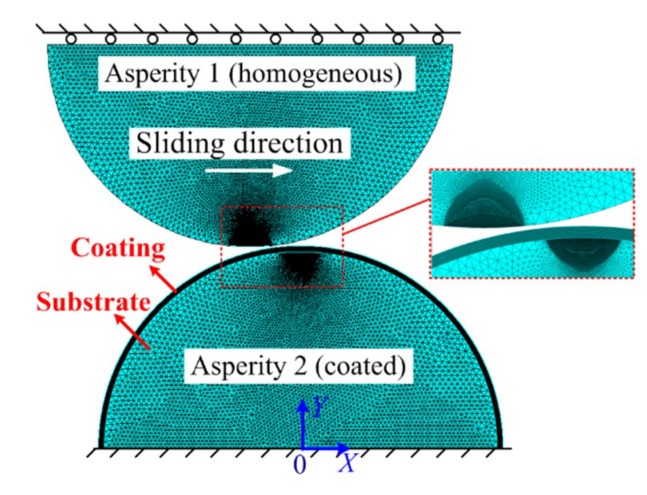
Finite element model.

A two-dimensional (2D) plane strain model was proposed to study the sliding interaction with the commercial finite element (FE) software ANSYS 17.0, as shown in Figure 2. The eight-node PLANE 183 element was used to mesh the asperities, and the contact was accomplished by the contact element (CONTA172) and the target element (TAEGET169). The PLANE 183 element is a higher-order 2D element, which could be employed as a plane element or as an axisymmetric element, having plasticity, hyperelasticity, creep, stress stiffening, large deflection, and large strain capabilities. In addition, the CONTA172 element is applicable to 2D structural and coupled-field contact analyses, representing a deformable surface and the TARGE169 element could represent 2D target surfaces. These two kinds of elements are used for both pair-based contact and general contact. The total number of the elements was 77,002. The geometrical and material parameters were set as shown in Table 1. The von Mises yield criterion was adopted, and the frictionless condition was used in the sliding process. Two boundary conditions were set: (a) The base of Asperity 2 was fixed in all directions and (b) the base of Asperity 1 could only move along the Y axis. The displacement load was applied to the base of Asperity 1 along the Y axis in a quasi-static way in the sliding process. The contact force was focused and computed step by step in this work. The mesh convergence was checked by doubling the mesh density iteratively until the change of the results was less than 2% between two consecutive steps.

**Figure 3:**
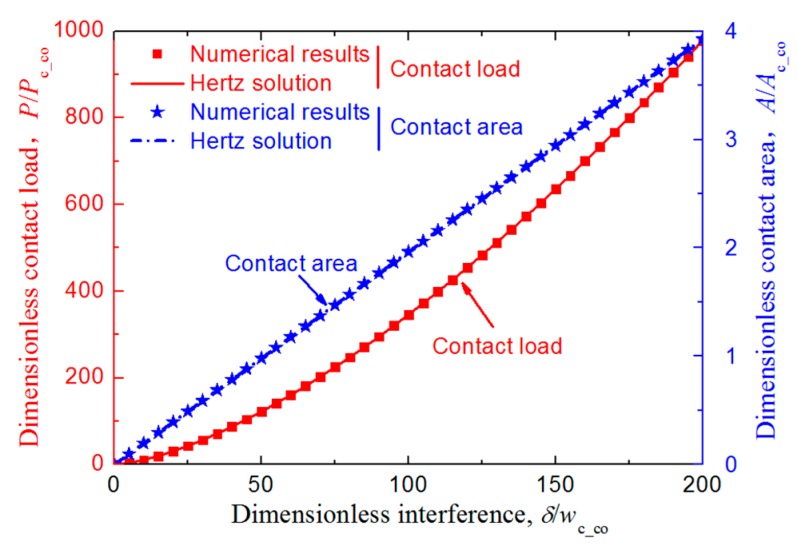
Comparison of the dimensionless contact parameters (including contact load, *P*/*P*_c_co_, and area, *A*/*A*_c_co_) between the Hertz results and the FE model developed in this work considering the purely elastic material cases when the two asperities were aligned vertically with respect to the axes of symmetry of Asperity 2 (*X* = 0) without sliding.

Besides the mesh convergence check, to ensure the validation of the developed FE model further, the relation between the dimensionless normal contact load, *P*/*P*_c_co_; the dimensionless contact area, *A*/*A*_c_co_; and the dimensionless interference, *δ*/*w*_c_co_, was considered for the contact between an elastic homogeneous asperity and an elastic coated asperity whose substrate and coating have the same materials (i.e., *E*_co_ = *E*_su_ = 200 GPa, *Y*_co_ = *Y*_su_ = 200 MPa) when the two asperities were aligned vertically with respect to the axes of symmetry of Asperity 2 (*X* = 0) without sliding. *P*_c_co_, *A*_c_co_, and *w*_c_co_ are the critical load, area, and interference of the coating material, which could be obtained by the equations given by Jackson and Green [28]:

(1) $$P_{c\_\mathrm{co}}=\frac{\pi^{3}Y_{\mathrm{co}}}{6}C_{v}{}^{3}{\left(R(1-v^{2})(\frac{Y_{\mathrm{co}}}{E’})\right)}^{2},$$

(2) $$A_{c\_\mathrm{co}}=\pi^{3}{\left(\frac{C_{v}Y_{\mathrm{co}}R}{2E’}\right)}^{2},$$

(3) $$w_{c\_\mathrm{co}}={\left(C_{v}\frac{\pi (1-v^{2})}{2}(\frac{Y_{\mathrm{co}}}{E’})\right)}^{2}R,$$

where *C_ν_* is the maximum dimensionless contact pressure at yielding inception related to the Poisson’s ratio, *v*, in slip condition satisfying *C_ν_* = 1.234 + 1.256*v* [28], and *E’* is the equivalent elastic modulus of the contact pair. The two asperities were in vertical contact along the axes of symmetry (i.e., *w*_h_ = 0 in Figure 1) with no sliding. The FE results were compared with the Hertz results as shown in Figure 3, and the difference of the contact force and area were both no more than 1.1%, which indicated the FE model was reliable.

**Figure 4:**
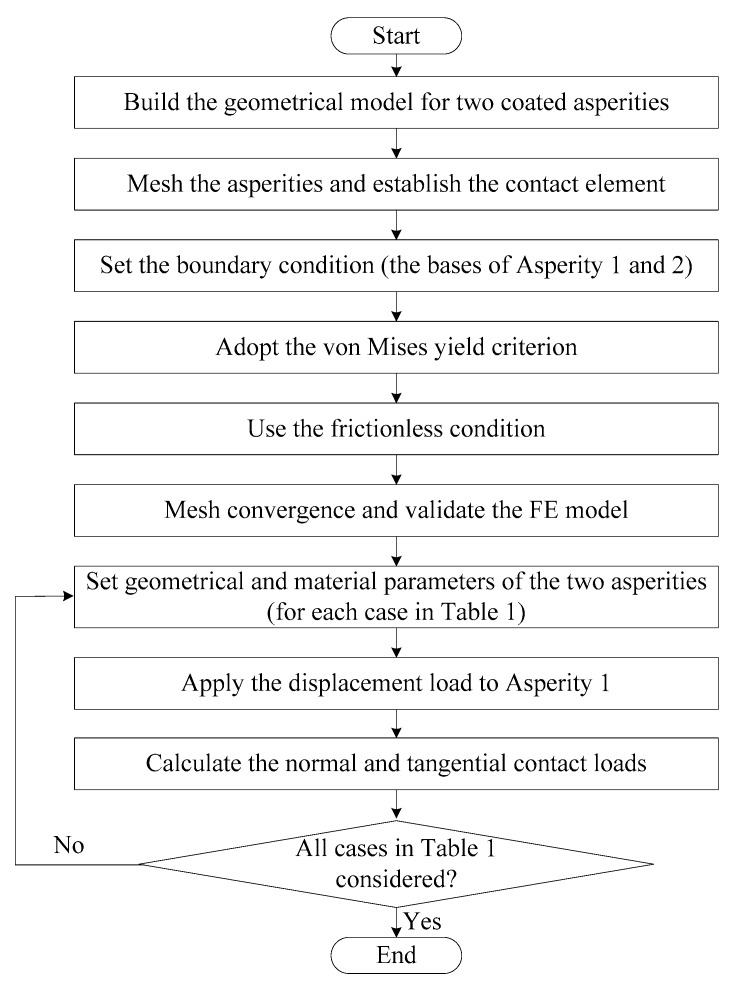
The flowchart showing the details of the development of the FE model.

In summary, the FE model is developed as shown in Figure 4. 

## 3. Results and Discussion

**Figure 5:**
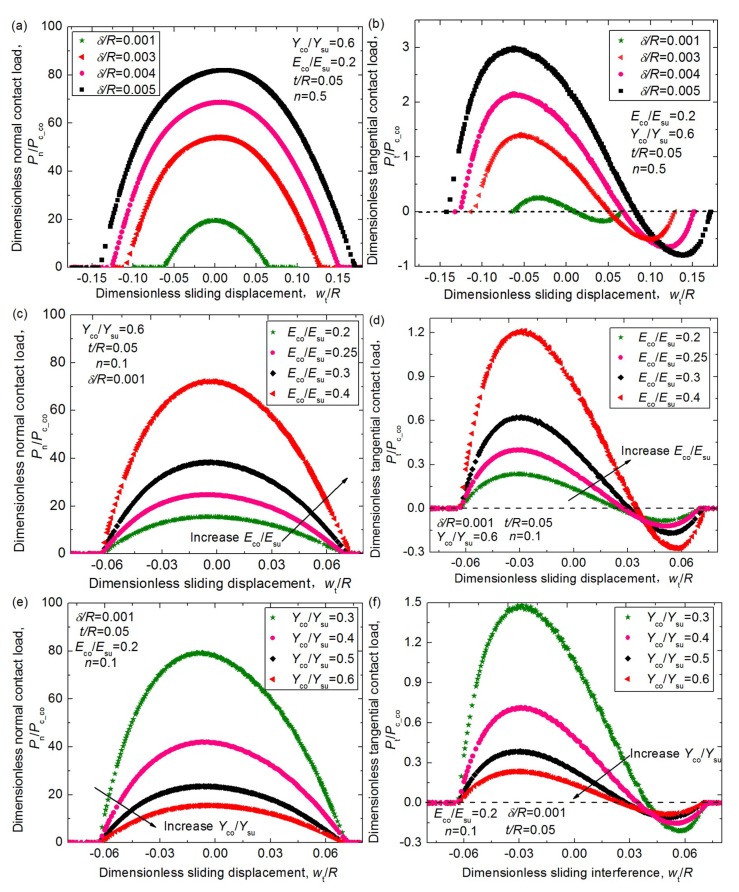

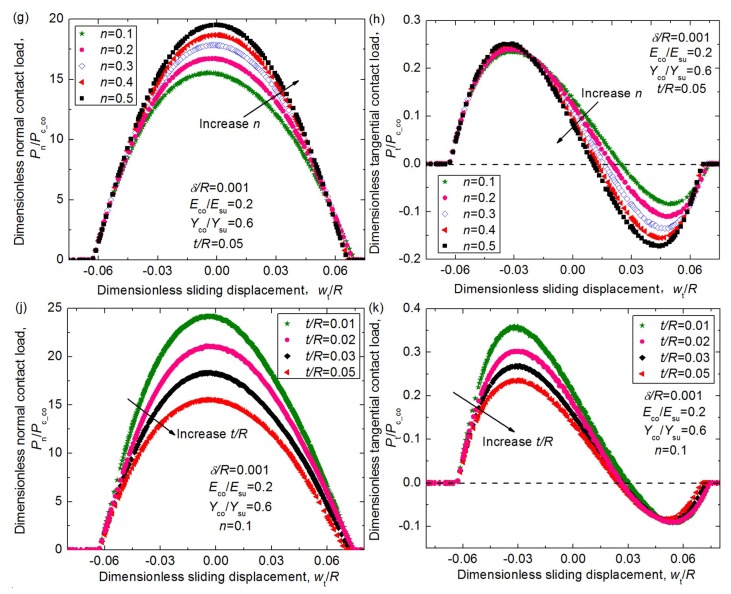
The relation of the dimensionless normal and tangential load, *P*_n_/*P*_c_co_, *P*_t_/*P*_c_co_, and the dimensionless interference, *w*_t_/*R*, under different normal interferences, *δ/R*, for different cases where Young’s modulus ratios, *E*_co_/*E*_su_, yield strength ratios, *Y*_co_/*Y*_su_, hardening exponent, *n*, and thickness, *t/R*, of the coating change in specific ranges. (**a**) The effect of *δ/R* on *P*_n_/*P*_c_co_; (**b**) The effect of *δ/R* on *P*_t_/*P*_c_co_; (**c**) The effect of *E*_co_/*E*_su_ on *P*_n_/*P*_c_co_; (**d**) The effect of *E*_co_/*E*_su_ on *P*_t_/*P*_c_co_; (**e**) The effect of *Y*_co_/*Y*_su_ on *P*_n_/*P*_c_co_; (**f**) The effect of *Y*_co_/*Y*_su_ on *P*_t_/*P*_c_co_; (**g**) The effect of *n* on *P*_n_/*P*_c_co_; (**h**) The effect of *n* on *P*_t_/*P*_c_co_; (**j**) The effect of *t/R* on *P*_n_/*P*_c_co_; (**k**) The effect of *t/R* on *P*_t_/*P*_c_co_.

Based on the developed FE model, the process where a homogenous asperity slides across a coated asperity was analyzed, and the relation between the dimensionless normal load, *P*_n_/*P*_c_co_; the tangential load, *P*_t_/*P*_c_co_; and the sliding displacement, *w*_t_/*R* was considered. The effect of the interference, *δ/R*; the Young’s modulus ratio, *E*_co_/*E*_su_; the yield strength ratio, *Y*_co_/*Y*_su_; the coating hardening exponent, *n*; and the coating thickness, *t/R*, on the normal and tangential loads was considered, and some typical results are shown in Figure 5. Figure 5a,b shows that the normal load and the tangential load get notably larger with the increase of the interference, *δ/R*, which is expected, since larger interference means the asperities deform more heavily and plastically. Moreover, for larger *δ/R* values, the tangential load reaches the maximum values earlier, then goes back to zero later, and the asperities show a more significant dragging effect in the process of moving away from each other. Figure 5c,d shows the effect of the Young’s modulus ratio, *E*_co_/*E*_su_, on the contact load. It reveals that larger *E*_co_/*E*_su_ values lead to larger normal and tangential loads under the same interference in the whole sliding process since the larger *E*_co_/*E*_su_ values represent the more elastic coatings. The maximum dimensionless normal loads for the *E*_co_/*E*_su_ = 0.4 case are about 4.6 times larger than the case where *E*_co_/*E*_su_ = 0.2, while the corresponding tangential loads are 5.1 times larger, which means the influence of the *E*_co_/*E*_su_ on the contact force is significant. Figure 5e,f shows the effect of the yield strength ratio, *Y*_co_/*Y*_su_. It could be seen that as the *Y*_co_/*Y*_su_ value increases from 0.3 to 0.6, the maximum dimensionless normal load, *P*_n_/*P*_c_co_, decreases notably from 79.3 to 15.5, and the maximum tangential load, *P*_t_/*P*_c_co_, decreases from 1.479 to 0.235. It seems to be not in line with the expectation as the normal load, *P*_n_, and tangential load, *P*_t_, should increase with the increase of the *Y*_co_/*Y*_su_ value under a special *E*_co_/*E*_su_ and normal interference *δ* case. The reason why *P*_n_/*P*_c_co_ and *P*_t_/*P*_c_co_ decrease is that the increase of the *P*_c_co_ value is greater than that of the *P*_n_ and *P*_t_ values as *Y*_co_/*Y*_su_ gets larger. Figure 5g,h demonstrates that the hardening exponent, *n*, also affects the dimensionless loads to some extent. As the *n* value increases from 0.1 to 0.5, the maximum normal loads increase about 25.8%. The maximum tangential loads before the vertical alignment axis (*w*_h_*/R* = 0) do not change so notably, while after the vertical alignment axis, the effect of the *n* values is revealed. For the smaller *n* cases, the tangential loads get smaller and the maximum values appear later since the coating is more plastic and the pile-up effect is more significant. Figure 5j,k demonstrates that as the coating thickness, *t/R*, gets larger, the normal load becomes smaller notably since the coating materials are softer than the substrate, and the tangential load also shows the same regularity before the axis (*w*_h_*/R* = 0). However, it seems that the coating thickness has little influence on the pile-up effect since the difference between the tangential loads after the axis *w*_h_*/R* = 0 are very small for different *t/R* cases.

**Figure 6:**
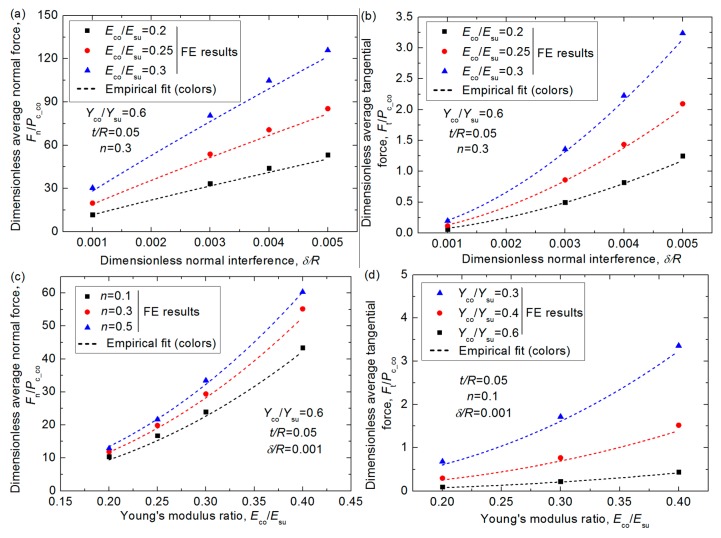

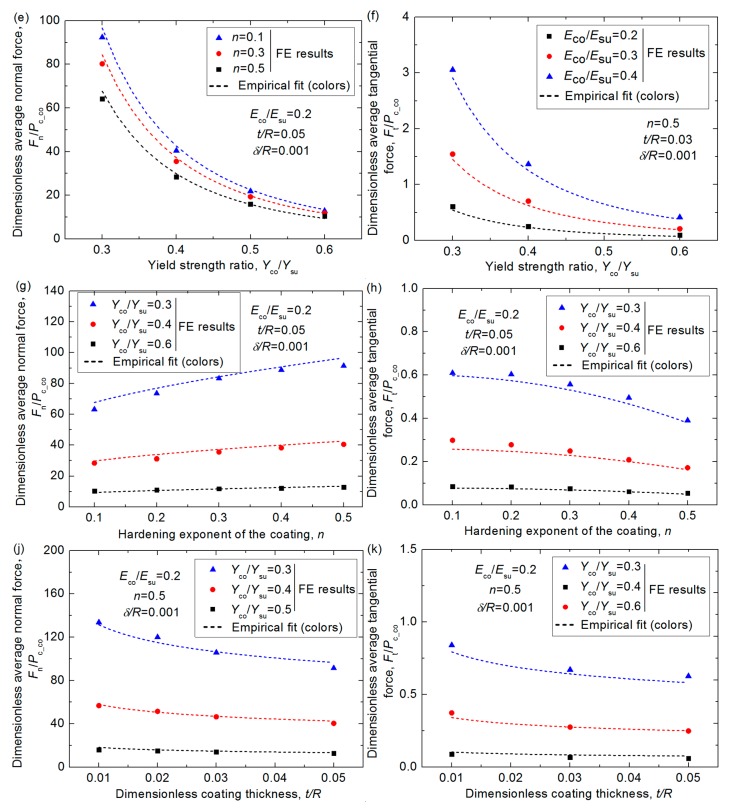
The dimensionless average normal and tangential force, *F*_n_/*P*_c_co_ and *F*_t_/*P*_c_co_, under different normal interferences, *δ/R*, for different cases where Young’s modulus ratios, *E*_co_/*E*_su_, yield strength ratios, *Y*_co_/*Y*_su_, hardening exponent, *n*, and thickness, *t/R*, of the coating changes in different specific range. (**a**) The effect of *δ/R* on *F*_n_/*P*_c_co_; (**b**) The effect of *δ/R* on *F*_t_/*P*_c_co_; (**c**) The effect of *E*_co_/*E*_su_ on *F*_n_/*P*_c_co_; (**d**) The effect of *E*_co_/*E*_su_ on *F*_t_/*P*_c_co_; (**e**) The effect of *Y*_co_/*Y*_su_ on *F*_n_/*P*_c_co_; (**f**) The effect of *Y*_co_/*Y*_su_ on *F*_t_/*P*_c_co_; (**g**) The effect of *n* on *F*_n_/*P*_c_co_; (**h**) The effect of *n* on *F*_t_/*P*_c_co_; (**j**) The effect of *t/R* on *F*_n_/*P*_c_co_; (**k**) The effect of *t/R* on *F*_t_/*P*_c_co_.

**Figure 7:**
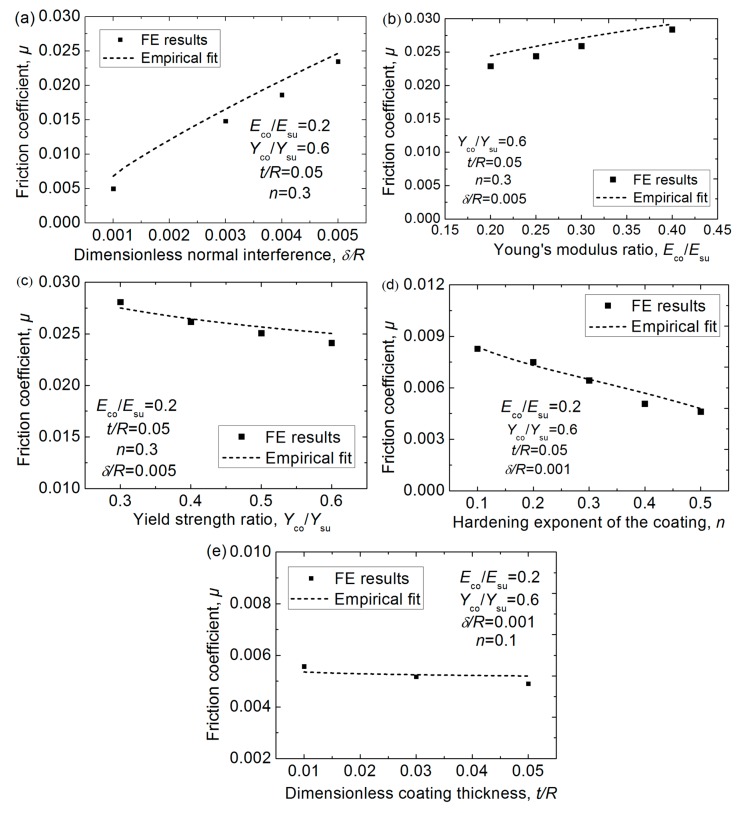
The effective friction coefficient due to the plastic deformation *μ* in the asperity sliding process under different normal interferences, *δ/R*, for different cases where Young’s modulus ratios, *E*_co_/*E*_su_, yield strength ratios, *Y*_co_/*Y*_su_, hardening exponent, *n*, and thickness, *t/R*, of the coating changes in a different specific range. (**a**) The effect of *δ/R* on *μ*; (**b**) The effect of *E*_co_/*E*_su_ on *μ*; (**c**) The effect of *Y*_co_/*Y*_su_ on *μ*; (**d**) The effect of *n* on *μ*; (**e**) The effect of *t/R* on *μ*.

Based on the results of the normal and tangential loads, the average dimensionless normal force, *F*_n_/*P*_c_co_, in the sliding process could be calculated with the following expression (see [18]):

(4) $$F_{n}/P_{c\_\mathrm{co}}=\frac{1}{{\left(w_{t}{}^{*}\right)}_{i}+{\left(w_{t}{}^{*}\right)}_{o}}\int_{{\left(w_{t}{}^{*}\right)}_{i}}^{{\left(w_{t}{}^{*}\right)}_{o}}f(w_{t}{}^{*})dw_{t}{}^{*},$$

where *w*_t_^*^ means the dimensionless expression of sliding displacement, *w*_t_/*R*, and the expression *f*(*w*_t_^*^) represents the relation of *P*_n_/*P*_c_co_ and *w*_t_^*^ as shown in Figure 5, which could be expressed approximately as high-order polynomials:

(5) $$f(w_{t}{}^{*})=a{\left(w_{t}{}^{*}\right)}^{5}+b{\left(w_{t}{}^{*}\right)}^{4}+c{\left(w_{t}{}^{*}\right)}^{3}+d{\left(w_{t}{}^{*}\right)}^{2}+e(w_{t}{}^{*})+g$$

(*w*_t_^*^)_i_ means the position where the asperities first come into contact, and (*w*_t_^*^)_o_ means the position where the asperities are out of contact with each other. The average dimensionless tangential force, *F*_t_/*P*_c_co_, could also be obtained in the same way for all cases in this work (see Table 1) by fitting with the finite element results. The effect of the material parameters, *E*_co_/*E*_su_, *Y*_co_/*Y*_su_, *n*, and the geometrical parameter, *t/R*, under different interferences, *δ/R*, on the average normal and tangential forces were considered, as shown in Figure 6. It can seen from Figure 6a,b that both the average normal force and tangential force increase with the sliding interference, *δ/R*, as expected since larger interference means more plastic deformation in the contact region when the material and the geometry of the contacting asperity keep unchanged. The increase is more significant for the tangential force, indicating the friction coefficient would be larger for larger *δ/R* cases, which is shown more clearly in Figure 7 later. Figure 6c–f shows the effect of the Young’s modulus ratio, *E*_co_/*E*_su_, and the yield strength ratio, *Y*_co_/*Y*_su_, on the normal and tangential forces, respectively, and it is shown the effect is notable in the focused ranges shown in Table 1. The larger *E*_co_/*E*_su_ and smaller *Y*_co_/*Y*_su_ values lead to larger average forces when other parameters are constant. The reason has been given as mentioned before. The hardening exponent, *n*, and the thickness, *t/R*, of the coating also have a certain effect on the average forces as displayed in Figure 6g–k. As *n* increases, the average normal force increases and the tangential force decreases, while as *t/R* increases, the average forces decrease gradually. It is because the coatings with larger *n* values mean the coated asperities are more elastic and the dragging effect is less significant, thus the normal force is larger, and the tangential force is smaller under the same condition. In addition, the thicker soft coating means the coated asperity is more plastic, thus, the normal force gets smaller. Furthermore, the average tangential force decreases as the coating thickness increases, it is because the tangential load gets smaller as the value of *t*/*R* increases before the vertical alignment axis (*w*_h_*/R* = 0) but it changes a little after the vertical alignment axis as indicated in Figure 5k.

Considering the effect of the material parameters, *E*_co_/*E*_su_, *Y*_co_/*Y*_su_, *n*, and the geometrical parameter, *t/R*, under different interferences, *δ/R*, the empirical expression of the average dimensionless normal force *F*_n_/*P*_c_co_ and tangential force *F*_t_/*P*_c_co_ could be derived as the following Equations (6) and (7):

(6) $$F_{n}/P_{c\_\mathrm{co}}=75.163{\left(E_{co}/E_{\mathrm{su}}\right)}^{2.168}{\left(Y_{co}/Y_{\mathrm{su}}\right)}^{-2.848}(206.469+298.274n^{0.5858}){\left(t/R\right)}^{-0.185}{\left(\delta /R\right)}^{0.907}$$

(7) $$F_{t}/P_{c\_\mathrm{co}}=429.929{\left(E_{co}/E_{\mathrm{su}}\right)}^{2.425}{\left(Y_{co}/Y_{\mathrm{su}}\right)}^{-2.936}(144.248-247.152n^{2.9}){\left(\delta /R\right)}^{1.702}{\left(t/R\right)}^{-0.203}$$

The comparison between some typical FE results and the predicted results given by Equations (6) and (7) is also shown in Figure 6. It shows the differences change from 1.2% to 8.3%, which means the fitting equations could express the FE results accurately.

The friction coefficient is an important parameter in the tribological field. In this work, the effective friction coefficient, *μ*, due to the plastic deformation in the asperity sliding process could be obtained using the average normal and tangential forces with Equations (6) and (7), shown here as Equation (8):

(8) $$\begin{array}{l} \mu =F_{n}/F_{t}\\ \;=5.72*{\left(E_{co}/E_{\mathrm{su}}\right)}^{0.257}*{\left(Y_{co}/Y_{\mathrm{su}}\right)}^{-0.088}{\left(\delta /R\right)}^{0.795}{\left(t/R\right)}^{-0.018}(144.248-247.152*n^{2.9})/(206.469+298.274n^{0.5858}) \end{array}$$

The comparison between the FE results and the empirical fitting results given by Equation (8) is shown in Figure 7, which reveals the fit is good. It should be noted that the friction coefficient given by Equation (8) only comes from the energy loss due to the plastic deformation, which is only one source of the friction. It could be seen that the value of *μ* increases with the increasing interference, *δ/R*, and Young’s modulus ratio, *E*_co_/*E*_su_, while it decreases as the yield strength ratio, *Y*_co_/*Y*_su_, the hardening exponent, *n*, and the thickness, *t/R*, of the coating increase. 

For the materials of these two asperities that are used widely in the journal bearing of the marine engine, the fitting expressions Equations (6)–(8) for the single asperity sliding process could be used to consider the average contact force, tangential force, and friction coefficient for rough surface contact. In addition, only the friction due to the plastic deformation was focused on in this work, since in some cases, this component of the friction dominates, while the adhesion was omitted here, but will be considered in the future.

## 4. Conclusions

The asperity sliding was inevitable in poor lubrication conditions where asperity contact exists. In this work, the sliding process between a coated asperity and a homogeneous asperity was studied to consider the frictional behavior of the crankshaft-bearing system in the marine engine on the micro scale. Some conclusions are given as follows:

(1) The contact parameters including the normal and tangential load, the average forces, and the effective friction coefficient were considered in the sliding process, and the corresponding universal expressions were obtained.
(2) The contact interference, the Young’s modulus ratio and yield strength ratio of the coating and substrate, and the hardening exponent and thickness of the coating show some influence on the contact parameters to some extent.
(3) The friction coefficient increases when the interference and Young’s modulus ratio enlarge, and it decreases as the yield strength ratio and the coating’s hardening exponent and thickness increase. This will help select the optimum coatings to reduce friction for the focused journal bearing in the marine engine.

## Figures and Tables

**Table 1:** Geometrical and material parameters.

Parameters	Asperity 1	Coating of Asperity 2	Substrate of Asperity 2
*t/R*	-	0.001–0.005	-
*δ/R*	0–0.005		
*E* (GPa)	200 (*E*_1_)	40–80 (*E*_co_)	200 (*E*_su_)
*Y* (MPa)	500 (*Y*_1_)	60–120 (*Y*_co_)	200 (*Y*_su_)
*v*	0.33 (*v*_1_)	0.33 (*v*_co_)	0.33 (*v*_su_)
Hardening type	2% linear hardening	*n* = 0.1–0.5 power-law hardening	2% linear hardening

## Nomenclature

A	Contact area
A_c_co_	Critical area of the coating material
E’	Equivalent elastic modulus of the contact asperity pair
E_1_	Young’s modulus of Asperity 1
E_co_	Young’s modulus of the coating of Asperity 2
E_su_	Young’s modulus of the substrate of Asperity 2
F	Contact force
F_n_	Average normal contact force
F_t_	Average tangential contact force
n	Hardening exponent of the coating of Asperity 2
P_c_co_	Critical load of the coating material
P_n_	Normal load
P_t_	Tangential load
R	Radius of the asperities
t	Coating thickness of the coated asperity
w_c_co_	Critical interference of the coating material
w_h_	Horizontal coordinate of Asperity 1
w_t_	Sliding displacement of Asperity 1
w_t_^*^	Dimensionless expression of sliding displacement of Asperity 1, w_t_/R
Y_1_	Yield strength of Asperity 1
Y_co_	Yield strength of the coating of Asperity 2
Y_su_	Yield strength of the substrate of Asperity 2
δ	Overlap of the two asperities
μ	Effective friction coefficient
ν_1_	Poisson’s ratio of Asperity 1
ν_co_	Poisson’s ratios of the coating of Asperity 2
ν_su_	Poisson’s ratios of the substrate of Asperity 2
